# Isolation of Dibutyl Phthalate-Degrading Bacteria and Its Coculture with *Citrobacter freundii* CD-9 to Degrade Fenvalerate

**DOI:** 10.4014/jmb.2110.10048

**Published:** 2022-01-14

**Authors:** Min Wu, Jie Tang, Xuerui Zhou, Dan Lei, Chaoyi Zeng, Hong Ye, Ting Cai, Qing Zhang

**Affiliations:** Key Laboratory of Food Biotechnology, College of Food and Bioengineering, Xihua University, Chengdu 610039, Sichuan, P.R. China

**Keywords:** DBP, fenvalerate, coculture, *Citrobacter freundii*, *Stenotrophomonas acidaminiphila*

## Abstract

Continued fenvalerate use has caused serious environmental pollution and requires large-scale remediation. Dibutyl phthalate (DBP) was discovered in fenvalerate metabolites degraded by *Citrobacter freundii* CD-9. Coculturing is an effective method for bioremediation, but few studies have analyzed the degradation pathways and potential mechanisms of cocultures. Here, a DBP-degrading strain (BDBP 071) was isolated from soil contaminated with pyrethroid pesticides (PPs) and identified as *Stenotrophomonas acidaminiphila*. The optimum conditions for DBP degradation were determined by response surface methodology (RSM) analysis to be 30.9 mg/l DBP concentration, pH 7.5, at a culture temperature of 37.2°C. Under the optimized conditions, approximately 88% of DBP was degraded within 48 h and five metabolites were detected. Coculturing *C. freundii* CD-9 and *S. acidaminiphila* BDBP 071 promoted fenvalerate degradation. When CD-9 was cultured for 16 h before adding BDBP 071, the strain inoculation ratio was 5:5 (v/v), fenvalerate concentration was 75.0 mg/l, fenvalerate was degraded to 84.37 ± 1.25%, and DBP level was reduced by 5.21 mg/l. In addition, 12 fenvalerate metabolites were identified and a pathway for fenvalerate degradation by the cocultured strains was proposed. These results provide theoretical data for further exploration of the mechanisms used by this coculture system to degrade fenvalerate and DBP, and also offer a promising method for effective bioremediation of PPs and their related metabolites in polluted environments.

## Introduction

Fenvalerate is a member of the type II synthetic or biomimetic pyrethroid pesticides (PPs) having broad-spectrum, highly efficacious and fast-acting properties with wide application in agriculture, forestry, and households [1-3]. However, long-term use of fenvalerate is hazardous to the environment and food chain and it has been reported that it may adversely affect fish, aquatic insects [4], beetles [5], bees [6] and microorganisms [7]. Moreover, studies have shown that fenvalerate is cumulative [8, 9] and long-term exposure can cause chronic diseases [10, 11]. Due to the potential of serious environmental pollution and ecological damage caused by the continuous increase in fenvalerate residues, the elimination of fenvalerate residues from food processing and the environment is urgent and important. One technique, bioremediation, holds true remediation for soil contaminated by PPs over a relatively short time period [11-14].

Pesticide metabolites have also been studied [15-18]. Previous studies on biodegradation products have mainly focused on 3-phenoxybenzoic acid (3-PBA) due to its high toxicity [18]. However, a number of other studies have recently reported that phthalate esters (PAEs) were detected in the biodegradation metabolites of PPs, which were obtained by diaryl cleaving of 3-phenoxybenzaldehyde [19-22], including 1,2-benzenedicarboxylic acid bis (2-methylpropyl) ester [13], 1,2-benzenedicarboxylic acid, dipropyl ester [19], 1,2-benzenedicarboxylic, butyl dacyl ester [20, 21], and dibutyl phthalate (DBP) [22]. Moreover, Wang *et al*. [23] showed that DBP residue reached 2.8 -14.6 mg/kg, in Mollisol regions. Liu *et al*. [24] found that the concentration of DBP in the soil reached 0.04 - 29.4 mg/kg, in China. Studies also have shown that DBP adversely impacts the development of the immune system [25] and the nervous system [26, 27], and can cause adult weight gain, obesity [28, 29], and diabetes [30]. Currently, DBP-degrading bacteria include *Enterobacter* [23], *Arthrobacter* [24], *Bacillus* [31, 32], *Rhodococcus* [33, 34], *Sphingomonas* [35], *Paracoccus* [36], and *Pseudomonas* [37]. To our knowledge, the degradation of DBP by *Stenotrophomonas acidaminiphila* has not been reported.

The accumulation of PPs and DBP in the environment represents a serious threat to human health and has roused public concern [23]. Most single microorganisms cannot completely mineralize PPs [38]. Several studies have described the degradation of PPs and their intermediate metabolite, 3-PBA, by single strains [20, 38-42]. However, there is no report of synergistic simultaneous degradation of PPs and the metabolite DBP by cocultured strains. Therefore, exploring the effects of cocultures of DBP-degrading bacteria and pyrethroid-degrading bacteria is of great significance.

We previously showed that *Citrobacter freundii* CD-9 had a good fenvalerate degradation ability, and poor ability to degrade DBP. Thus, in this study, our objectives were as follows: (I) isolate and characterize the DBP-degrading strain, BDBP 071, from soil contaminated with PPs, (II) optimize culture conditions for degradation of DBP, and (III) identify the intermediate metabolites produced by the degradation of fenvalerate by *S. acidaminiphila* BDBP 071 and *C. freundii* CD-9 cocultures.

## Materials and Methods

### Chemicals and Media

DBP (98.0%) was purchased from TCI Reagent Factory (China). Fenvalerate (96.0%) was purchased from Rongcheng Chemicals (China). Chromatographic-grade methanol, dichloromethane and acetonitrile were obtained from Sigma-Aldrich Reagent Co. (China). Other chemicals were of analytical grade and purchased from Kelong Chemical Co. (China).

Liquid and solid mineral salt medium (MM) and Luria-Bertani (LB) medium were prepared as described previously [1]. Fenvalerate and DBP were dissolved in acetonitrile to make a 10 g/l stock solution concentration, which was diluted using culture medium to achieve the desired working solution concentrations.

### Enrichment, Isolation, and Identification of DBP-Degrading Strain

Soil samples were collected from the tomato root soil that had been contaminated with PPs in Sichuan, China, and an enrichment culture technique to isolate DBP-degrading strains was used [14]. Enrichment and separation procedures were conducted as previously described [1]. Briefly, 1 g soil sample was added to a 250 ml Erlenmeyer flask containing 50 ml sterilized LB/MM medium and 25 mg/l DBP. The initial enrichment culture flask was incubated at 30°C and 180 rpm for 5 days. Subsequently, 5 ml medium aliquots were transferred to new Erlenmeyer flasks containing 50 ml LB/MM medium and different DBP concentrations (50, 100, 200, 400, 800, 1,600 mg/l). After six rounds of culturing, the final suspensions were diluted, plated, and incubated on MM agar plates containing 50 mg/l DBP [1] to obtain a single colony using DBP as the sole carbon and energy source. The degradation ability of the isolates was confirmed using high-performance liquid chromatography (HPLC). The highest DBP-degrading strain BDBP 071 was selected for further study.

Strain BDBP 071 was successively identified by colony morphology, Gram staining, cell morphology, physio-biochemical tests, and 16S rRNA sequence analysis. The 16S rRNA gene was amplified using the universal primers EU27F and 1490R [1]. PCR cycling conditions were according to the previous description [22], and the PCR product was sent to Tsingke Biological Technology Co. (China) for DNA sequencing. Sequence similarity was analyzed using the NCBI’s BLAST program (https://blast.ncbi.nlm.nih.gov/Blast.cgi) and the sequence was deposited in GenBank. MEGA 7.0 software was used to construct the phylogenetic tree.

### Inoculum Preparation

Strains CD-9 (GenBank Accession No. MN629225.1, Collection No. CGMCC 20106) and BDBP 071 (GenBank Accession No. MW281770; Collection No. CGMCC7.422) were stored at -50°C in 20% glycerol. Before the experiment, strains were thawed and inoculated in 100 ml Erlenmeyer flasks containing 25 ml sterile LB medium and incubated for 12 h at 37°C and 180 rpm [13]. After incubation, the culture solution was centrifuged (8, 000 ×*g*, 5 min at 4°C) and the bacterial cells were collected. The cell precipitate was washed three times with 0.85% sterile saline solution and resuspended in sterile saline solution to adjust the OD_600_ to approximately 1.0 in order to prepare a liquid inoculum [22] which was used in DBP and fenvalerate biodegradation studies.

### Growth and Degradation Curves of Strain BDBP 071

Strain BDBP 071 was cultivated in liquid LB medium containing 25 mg/l DBP. Then, 6% (v/v) bacterial solution was added to the medium, and non-inoculated medium was used as the control. The experiments were conducted in triplicate. The biodegradation was performed for 3 days at 37°C and 180 rpm. The growth OD_600_ of the strain was monitored using a UV-spectrophotometer and the residual amount of DBP was determined using HPLC [22]. The growth kinetic equation of strain BDBP 071 and the first-order degradation kinetic equation of DBP were in accordance with equation 1 and equation 2, respectively:

$$X=X_{0}e^{\mu }{{}_{m}}^{t}/[1-(X_{0}/X_{m})(1-e^{\mu }{{}_{m}}^{t})]$$ (1),

where *X* represents the cell concentration (OD_600_) at time *t*, *X*_0_ represents the initial cell concentration (OD_600_), *μ_m_* represents the maximum specific growth rate (h^−1^), *t* represents the culture time (h), and *X_m_* represents the maximum cell concentration (OD_600_).

$$C_{t}=C_{0}\times e^{-kt},t_{1/2}=\ln2/k$$ (2),

where *C_t_* is the DBP concentration at time *t* (mg/l), *C_0_* is the initial DBP concentration (mg/l), *k* is the DBP degradation rate constant (h^−1^), *t* means the degradation time (h), and *t_1/2_* indicates the half-life of DBP.

### Optimization of DBP Biodegradation Conditions

The optimal conditions for DBP biodegradation by strain BDBP 071 were confirmed using RSM [1]. Based on the results of single-factor experiment (temperature, pH, inoculum volume, and DBP concentration) [13], critical factors of temperature (34-40°C), pH (6.5-7.5) and DBP concentration (15-35 mg/l) were selected as independent variables. Subsequently, using Box-Behnken design, 17 experiments were carried out to build quadratic models. Each treatment included three replications. An equivalent volume of sterile saline solution served as a blank control. Data were analyzed using the Design-Expert software (version 10.0, USA) and a secondary model was constructed.

### Degradation of Fenvalerate by Strain Cocultures

Suspensions of strains CD-9 and BDBP 071 were prepared as described in Materials and Methods. The total inoculum volume of 6% (v/v) was cultured in LB liquid medium containing 100 mg/l concentration of fenvalerate. The effects of inoculation sequence, inoculation proportion of strains CD-9 and BDBP 071, and fenvalerate concentration on the degradation of fenvalerate were studied. To obtain the strain inoculation sequences, strain CD-9 was first inoculated in LB medium for 0, 4, 8, 12, 16, 20, and 24 h, before inoculating strain BDBP 071. Subsequently, under the optimal inoculation sequences of strains CD-9 and BDBP 071, the efficiencies of different strain ratios (10:0, 9:1, 8:2, 7:3, 6:4, 5:5, 4:6, 3:7, 2:8, 1:9, 0:10) in fenvalerate degradation were investigated. In addition, the degradation of different concentrations of fenvalerate (5, 25, 50, 75, 100, and 200 mg/l) by the cocultured strains was also explored. Each treatment was performed in triplicate, and a separate sample without the strains served as a control.

### Identification of Metabolites

Strain BDBP 071 (6.0%, v/v) was inoculated into LB medium containing 30 mg/l DBP. Strains CD-9 (3.0%, v/v) and BDBP 071 (3.0%, v/v) were added to LB medium containing fenvalerate (75 mg/l) and incubated at 37°C and 180 rpm. After 48 h, 20 ml of the culture sample was used to identify intermediate metabolites, and the metabolites were extracted using the procedure based on a previous GC-MS study by Tang *et al*. [22].

### HPLC Conditions and Analysis

The strains were inoculated in LB liquid medium containing different concentrations of fenvalerate (DBP) to determine their degradation abilities. Extraction and detection of residual fenvalerate (DBP) were consistent with previous results described by Tang *et al*. [22] and Zhang *et al*. [31]. Fenvalerate (10 g/l) and DBP (10 g/l) were accurately diluted using acetonitrile to give a series of standard solutions with concentrations ranging from 1.0 mg/l to 200.0 mg/l. Concentrations of fenvalerate and DBP were analyzed using a Waters 2695 (Waters, USA) equipped with a ZORBAX eclipse plus C_18_ column (4.6 mm × 150 mm × 5 μm). Fenvalerate and DBP concentrations were quantified according to the retention time (RT) and peak area of the standards. The fenvalerate and DBP degradation rates were calculated according to the equation below:

$$\text{Degradation rate(\%) = }(1-C/C_{0})\times 100\%$$ (3),

where *C* and *C_0_* represent the fenvalerate (DBP) content in inoculated and non-inoculated medium, respectively.

### GC-MS Conditions and Analysis

DBP and fenvalerate intermediates were identified using a Shimadzu GC2010 Plus gas chromatograph coupled to a Shimadzu MS2010 Plus mass spectrometer in electron ionization mode (70 eV) with a DB-5 capillary column (30.0 m × 0.25 mm × 0.25 mm). The operating conditions were as follows: the injection volume was l μl; injection mode was splitless at 250°C ; the temperatures of the transmission line and the ion source were 250°C and 280°C, respectively; helium (99.999%) was used as a carrier gas at a flow rate of 1.5 ml/min. The detecting conditions of DBP and fenvalerate followed the procedures of Sun *et al*. [23] and Tang *et al*. [22], respectively. The identification process was conducted three times. Compounds were identified by comparing the mass spectrum of each peak with those of authentic standards in a mass spectra library database (NIST, USA).

### Statistical Analysis

Statistical analysis of DBP degradation was conducted in Origin software (version 8.5). All experiments were conducted in triplicate, with no-inoculation conditions as the control, and the data were expressed as mean ± SD.

## Results

### Isolation and Identification of DBP-Degrading Bacteria

In this study, five strains with high DBP degradation ability were obtained: strain BDBP 015, strain BDBP 037, strain BDBP 058, strain BDBP 071, and strain BDBP 092. Strain BDBP 071 showed the highest degradation ability in liquid LB medium containing 25 mg/l DBP, degrading 58.25 ± 2.31% of DBP within 48 h. Hence, it was selected as an ideal strain for degrading DBP. The strain was grown on LB plates containing 25 mg/l DBP, producing round and yellow, opaque, smooth colonies with neat edges. Scanning electron microscopy (SEM) [22] was used to observe the morphology of strain BDBP 071 (Fig. S1) and showed that BDBP 071 strain is short and rod-shaped with varying lengths and is arranged individually or in pairs. Physio-biochemical experiments showed that the strain was a gram-negative bacillus, with positive catalase, oxidase, and V-P tests but with negative indole, sportiness, hydrogen sulfide, starch hydrolysis, gelatin liquefaction, ornithine decarboxylase, lysine decarboxylase, and M.R tests (Table S1).

**Fig. 1:**
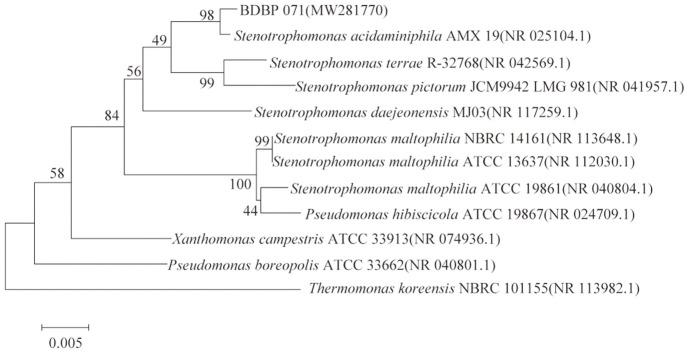
Phylogenetic tree of the strain BDBP 071 constructed by the neighbor-joining method based on 16S rRNA sequences of BDBP 071 and related strains. The numbers at the nodes represent the bootstrap value. Bar represents sequence divergence.

The BLAST search results showed that the 1446 bp sequence of the 16S rRNA gene from strain BDBP 071 shared 98% similarity with *Stenotrophomonas acidaminiphila* strain AMX 19 (Fig. 1) and the two strains clustered in the same clade in the phylogenetic tree. Based on the physiological and biochemical tests and 16S rRNA gene phylogenetic analysis, strain BDBP 071 was proposed to belong to *S. acidaminiphila*.

### Growth and Utilization of DBP by *S. acidaminiphila* BDBP 071

**Fig. 2:**
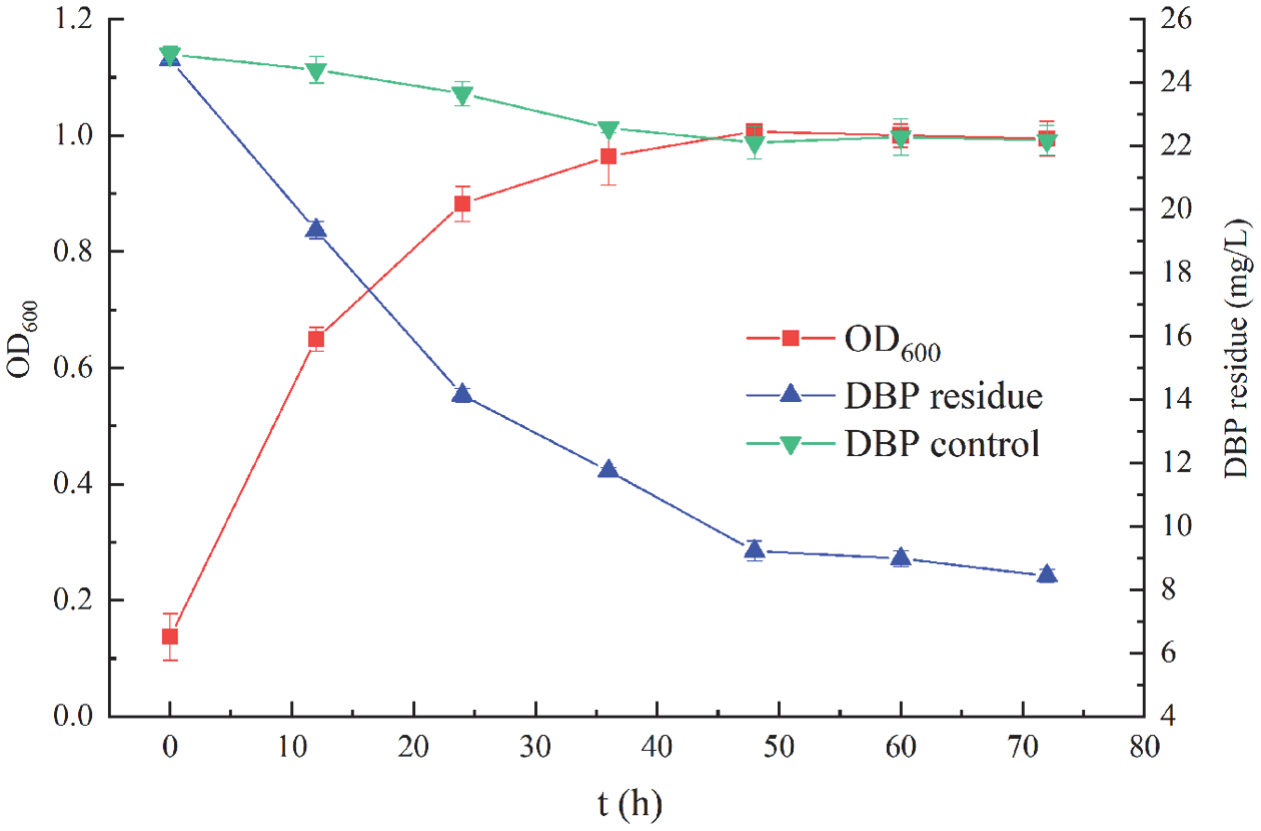
Degradation of DBP (25 mg/l) during growth of *S. acidaminiphila* BDBP 071. Error bars indicate standard deviation of three replicates.

The dynamic relationship between the growth of strain BDBP 071 in LB medium containing 25 mg/l DBP and DBP degradation rate was shown in Fig. 2. The OD_600_ of strain BDBP 071 rose from 0.1374 ± 0.04 to 1.0068 ± 0.01. The growth kinetics equation (Eq. 1) of strain BDBP 071 was as follows: X_BDBP 071_ = 0.17694e^0.17467*t*^/[1-0.17666 × (1-e^0.17467*t*^)], R^2^ = 0.9880; among them *μ_m_* = 0.17467 h^-1^, *X_0_* = 0.17694, *X_m_* = 1.00158. The first-order degradation kinetics model (Eq.2) was used to nonlinearly fit the DBP residues in the degradation process. Within 72 h, close to 60% of the 25 mg/l DBP initially added to the medium had been degraded by strain BDBP 071 and a first-order degradation kinetics equation was obtained as follows: *C*_BDBP 071_ = 23.94383e^-0.01851*t*^, *k* = 0.01851; *t_1/2_* = 37.45 h, R^2^ = 0.9523.

It is worth noting that the biodegradation of DBP started rapidly at the beginning of the strain BDBP 071 incubation, without an apparent lag phase. Degradation of DBP was associated with the growth of strain BDBP 071. At logarithmic phase (0-24 h), the growth of strain BDBP 071 increased rapidly, and significant degradation of DBP was noted (nearly 40.0% of the DBP was degraded). Subsequently, strain BDBP 071 grew slowly at stationary phase (24-60 h) while the density of strain BDBP 071 increased to its maximum level within 48 h of incubation and approximately 60.0% of the DBP was degraded during this period. After 72 h incubation, the residual amount of DBP did not change noticeably. No significant change in DBP concentration was observed in the non-inoculated controls.

### Optimization of Conditions for DBP degradation by Strain *S. acidaminiphila* BDBP 071

The single-factor test results showed that the concentration of DBP (5-200 mg/l), pH (4-12), and culture temperature (25-40°C) had significant effects on the degradation of DBP by *S. acidaminiphila* BDBP 071 (data not shown). Accordingly, optimizing these parameters improved the bacteria’s efficiency at degrading DBP. The interaction and effects of these three variables, observed with the help of 17 different experimental models, were presented in Table S2. The culture samples were collected at 48 h, when the highest and lowest degradation values of 87.56 ± 2.26% and 51.06 ± 1.27% were recorded. The data were analyzed using RSM and the quadratic model was employed to analyze DBP degradation. Subsequently, the experimental values obtained were fitted with the second-order polynomial equation (Eq. 4):

DBP degradation (%) = 78.58 + 6.64 pH + 2.13 temperature + 2.23 DBP concentration + 3.45 pH × temperature + 3.94 pH × DBP concentration - 5.64 temperature × DBP concentration - 0.20 pH^2^ - 12.41 temperature^2^ - 4.47 DBP concentration^2^ (4).

Results of analysis of variance (ANOVA) for DBP degradation showed that the model was a good fit (Table 1). The R^2^ (0.9841) and the R^2^_Adj_ = 0.9638 values were close to 1, indicating that the values predicted by the model were consistent with the experimental values. The high *F* value (*F* = 48.29) and extremely low *p*-value (*p* < 0.0001), show that the model had a significant regression and the equation could correctly reflect the relationship between DBP degradation and various factors. ANOVA of the regression equations showed that square terms of temperature and DBP concentration, and interaction terms of pH × temperature, pH × DBP concentration, temperature × DBP concentration, had significant effects (*p* < 0.05) on DBP degradation by strain BDBP 071, whereas pH^2^ played an insignificant role (*p*>0.05) in degradation.

**Fig. 3:**
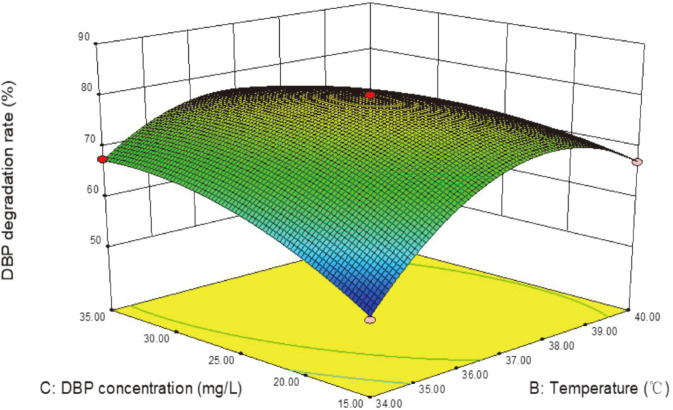
Three-dimensional plot showing the effects of (**B**) temperature and (**C**) DBP concentration on DBP degradation by strain BDBP 071. While fixing the (**A**) pH at the 1-coded level (pH 7.5).

The regression equation was graphically represented on a 3D response surface plot (Fig. 3). The plot had the steepest curved surface, indicating that the interaction between temperature and DBP concentration had the most significant impact on DBP degradation. The response surface plots showed the interaction of three parameters in DBP biodegradation. As a result, the optimized culture conditions for DBP degradation were pH 7.5, temperature of 37.0°C, and DBP concentration of 30.0 mg/l. Under optimal conditions, the degradation of DBP could reach 88.34 ± 1.44%, which is in agreement with the model prediction value (87.21%). The degradation rate of DBP increased by 30.09% after optimization, indicating that the design of the optimization scheme for degradation conditions is reasonable and effective.

### Degradation of Fenvalerate by Strain Cocultures

**Fig. 4:**
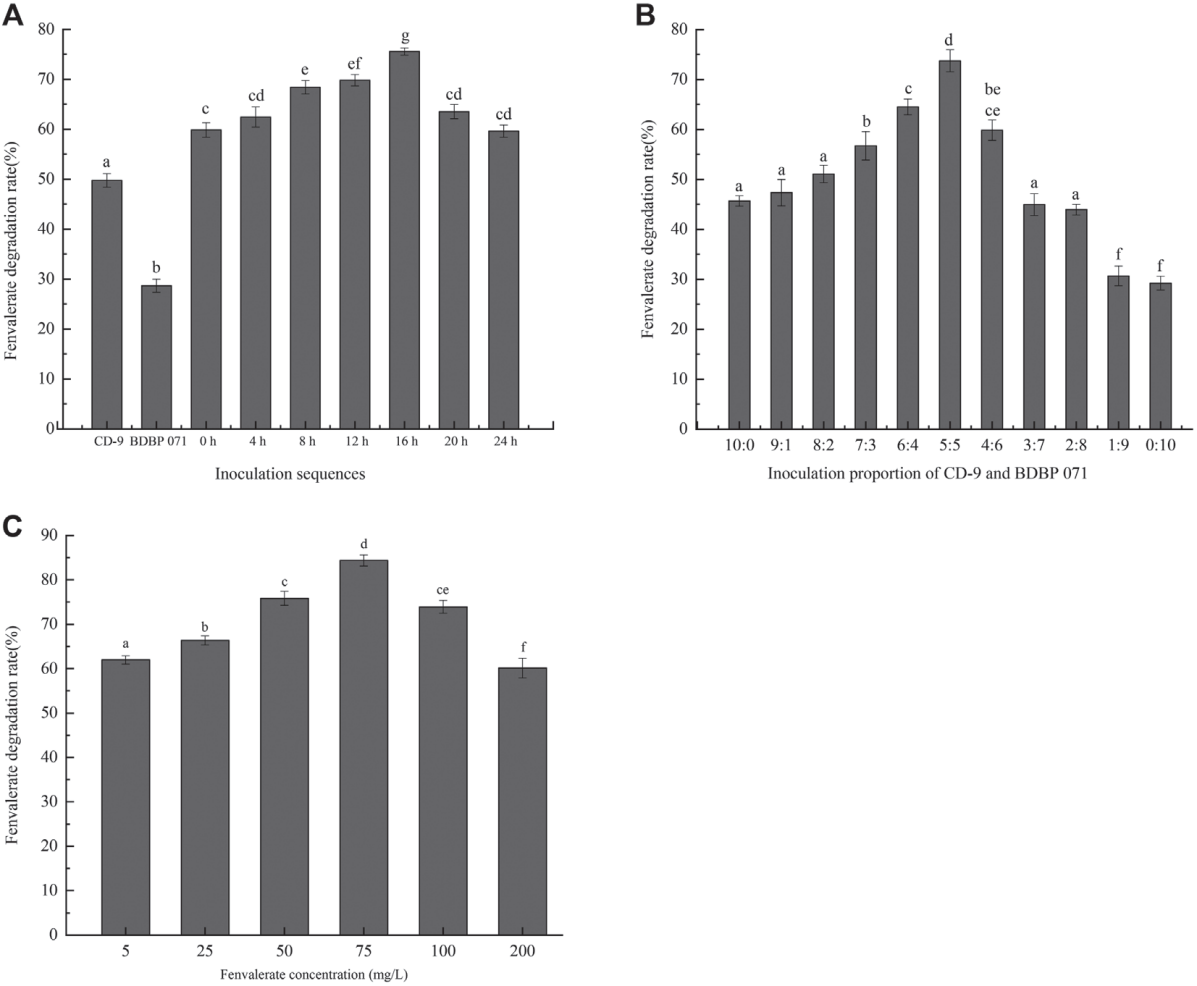
Effect of different culture conditions on the degradation of fenvalerate. (**A**) inoculation sequences (0 h, 4 h, 8 h, 12 h, 16 h, 20 h, 24 h indicate that the strain CD-9 was cultured for 0 h, 4 h, 8 h, 12 h, 16 h, 20 h, 24 h, and then the strain BDBP 071 was added), (**B**) inoculation proportions of strains CD-9 and BDBP 071, and (**C**) fenvalerate concentration. Different letters (a-g) indicate significant differences among treatments (*p* < 0.05).

Fig. 4 shows the results of the degradation of fenvalerate by a co-culture of strains CD-9 and BDBP 071. In the culture solution of strains synergistically degrading fenvalerate, the time of inoculation of strain BDBP 071 had a greater impact on the degradation of fenvalerate. For example, when only strain CD-9 was inoculated in the media, nearly 50% fenvalerate was degraded (Fig. 4A). However, when only strain BDBP 071 was used, only approximately 30% fenvalerate was degraded, indicating that strain BDBP 071 possesses poor fenvalerate degradation ability. When strain CD-9 was cultured for 4, 8, 12, 16, 20 h, and when strain BDBP 071 was then added to the culture, the degradation rate of fenvalerate surpassed 60% within 48 h. In particular, the degradation rate of fenvalerate was most obviously improved when strain CD-9 was first cultured for 16 h and then strain BDBP 071 added, reaching 75.57 ± 0.73%. The experimental conditions were that the concentration of fenvalerate was 100 mg/l and the strain inoculation ratio was 5:5 (6%, v/v).

Fenvalerate degradation using 11 different inoculation proportions of strains are shown in Fig. 4B. When the inoculation proportion of strains CD-9 and BDBP 071 ranged from 6:4 to 4:6, the strains could effectively enhance the degradation rate of fenvalerate and maximum fenvalerate degradation (73.75 ± 2.19%) was observed when the inoculation proportion was 5:5. In this experiment, the concentration of fenvalerate was 100 mg/l, and the inoculation sequence used was that strain CD-9 was first cultured for 16 h before adding BDBP 071. Degradation rates for various concentrations of fenvalerate (5, 25, 50, 75, 100, and 200 mg/l) were obtained under conditions of optimal inoculation sequence and inoculation ratio (Fig. 4C). The degradation rate of different concentrations of fenvalerate by the synergistic strains exceeded 60%. In particular, the degradation rate of 75.0 mg/l fenvalerate reached 84.37 ± 1.25%.

**Fig. 5:**
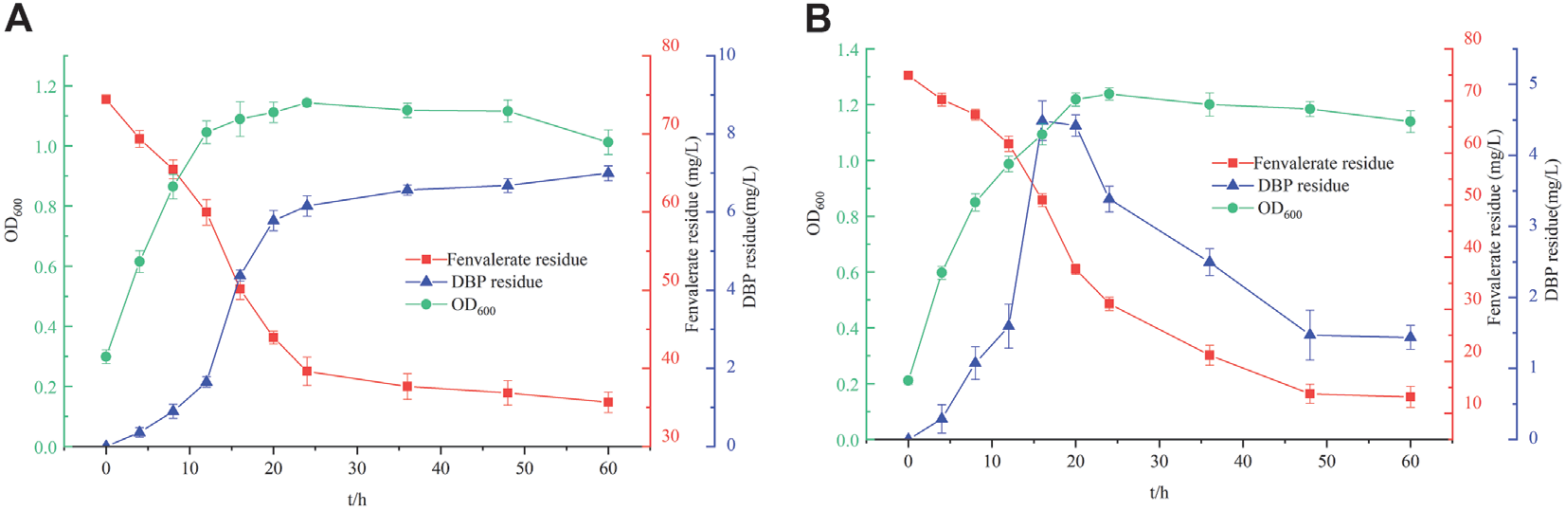
Degradation curve of fenvalerate. (**A**) Growth and fenvalerate degradation curve of *C. freundii* CD-9. (**B**) Variation in fenvalerate degradation and DBP content by using coculture of strains CD-9 and BDBP 071.

Temporal changes in microbial biomass (OD_600_), fenvalerate degradation, and DBP content in 75 mg/l fenvalerate following degradation by *C. freundi* CD-9 are shown in Fig. 5A. The amount of DBP produced increased with the increase in the degradation rate of fenvalerate, reaching a maximum value within 12 - 20 h. The degradation rate of fenvalerate was 50.87 ± 1.58% after 48 h of culturing, while the yield of DBP reached 6.68 ± 0.28 mg/l. The degradation rate did not increase significantly when the culturing was continued beyond this point. Strain CD-9 could degrade low concentrations of DBP (Fig. S2). When the DBP concentration was 5 mg/l, the degradation rate was only 31.96 ± 1.53%. However, as the DBP concentration increased, the degradation rate dropped rapidly and when DBP concentration was 100 mg/l, the degradation rate was 2.03 ± 1.31%. This demonstrates that strain CD-9 has poor DBP degradation ability.

Cocultures of strains CD-9 and BDBP 071 were used in the fenvalerate degradation process to enhance the degradation of fenvalerate and decrease the amount of the metabolite, DBP. Here, when strain CD-9 was cultured for 16 h before adding strain BDBP 071, the strain inoculation ratio was 5:5 (v/v), and the concentration of fenvalerate was 75.0 mg/l. Thus, the degradation of fenvalerate increased by 33.5%, and the level of DBP reduced by 5.21 mg/l (Fig. 5B) compared with the degradation of monoculture strain CD-9.

### Identification of DBP and Fenvalerate Biodegradation Metabolites

**Fig. 6:**
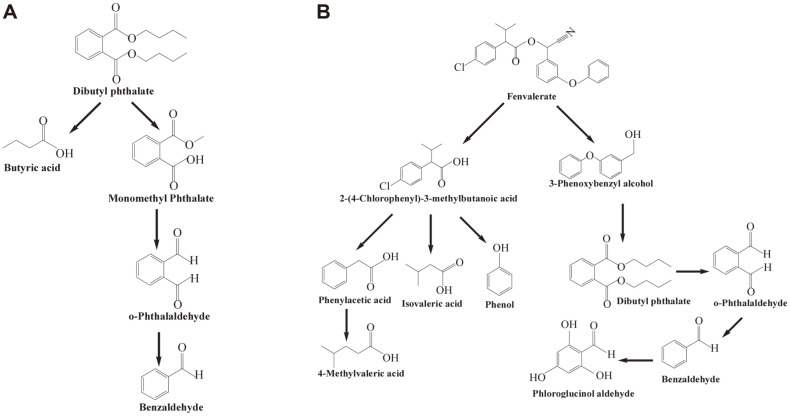
Proposed biodegradation pathway of DBP and fenvalerate. (**A**) Proposed biodegradation pathway of DBP by BDBP 071. (**B**) Proposed biodegradation pathway of fenvalerate by cocultures of strains CD-9 and BDBP 071.

To explore the mechanism of DBP degradation by *S. acidaminiphila* BDBP 071, the metabolites generated by DBP degradation were identified using GC-MS. After 48 h incubation, five main degradation products: butyric acid (a), O-phthalaldehyde (b), benzaldehyde (c), mono-methyl phthalate (MMP, d) and DBP (e) were identified. The retention time (RT), similarity, chemical formula, characteristic ions of the mass spectra (m/z), and names were summarized in Table 2. The possible metabolic pathway of DBP in strain BDBP 071 was proposed based on the metabolites (Fig. 6A). The enzymes of alkyl ester bond hydrolysis and oxygenase played a critical role in the biodegradation process of DBP [23, 36]. In the degradation pathway of DBP, DBP is hydrolyzed by β-oxidation and de-esterification to produce MMP and n-butanol [44]. Subsequently, butyric acid is generated from n-butanol [45] and MMP is metabolized to phthalic acid (PA) by demethylation, and then further through β-oxidation before entering the TCA cycle. However, in this article, PA was not detected, while O-phthalaldehyde was found, which may provide a new biodegradation pathway for DBP.

The potential metabolic products formed during fenvalerate co-degradation by strains CD-9 and BDBP 071 were also detected using GC-MS (Table 3). Compounds A – L were identified as isovaleric acid (**A**), benzaldehyde (**B**), phenol(**C**), O-phthalaldehyde (**D**), 4-methylhexanoic acid (**E**), phenylacetic acid (F), anthranilic acid (G), 2-(4-chlorophenyl)-3-methylbutanoic acid (H), 3-phenoxybenzyl alcohol (I), dibutyl phthalate (J), phloroglucinol aldehyde (K), and fenvalerate (L). A potential biodegradation pathway of fenvalerate by strains CD-9 and BDBP 071 was proposed based on the metabolites obtained (Fig. 6B). In it, compounds G and K have not been reported in the biodegradation process of fenvalerate. However, 3-aminobenzoic acid has been detected in the biodegradation products of beta-cypermethrin [46]. Moreover, 3,4,5-trihydroxybenozic acid was detected in the products obtained from the degradation of 3-PBA by strain M-4 [47]. These degradation products were slightly different from those reported previously [22]. In the proposed biodegradation pathway, the ester linkage is broken by hydrolase and the fenvalerate (L) is decomposed into carboxylic acid (H) and alcohol (I), which is the core step in the biodegradation of PPs [1]. Under the hydrolysis of halogen elements, compound H is converted to compounds F, A and C. Then the benzene ring of compound F is cleaved to produce compound E. Meanwhile, compounds C and J are generated from compound I through diaryl cleavage [13]. Then, compound D is formed by hydrolyzing compound J and converted into compounds B and K by oxygenase, decarboxylase, oxidoreductase, etc., possibly.

## Discussion

Microbial remediation of organic pollutants and pesticides has received significant research attention in recent years [7, 48]. In this study, a highly effective DBP-degrading strain, BDBP 071, was isolated from soil contaminated by PPs. Strain BDBP 071 was identified as *S. acidaminiphila* based on its morphological and biological characteristics, as well as the sequence of its 16S rRNA gene. *Stenotrophomonas* sp. was previously reported to degrade organophosphorus pesticides [49], chlorothalonil [50], butachlor [51], and aflatoxin B1 [52]. To our knowledge, this is the first research reporting on DBP degradation by *S. acidaminiphila*.

RSM was used to optimize degradation conditions [19]. At 37°C, 30.0 mg/l substrate concentration, and pH 7.5, strain BDBP 071 could degrade 88.34 ± 1.44% of 30.0 mg/l DBP in LB medium within 48 h. Several studies have reported that some bacterial strains are capable of degrading DBP [12, 21, 53, 54]. The ability of strain BDBP 071 to degrade DBP in this study is comparable to those of previously reported strains. For example, *Providencia* sp. 2D could degrade 84.9% of 1,000 mg/l DBP within 72 h [55], 89.74% of 5 mg/l DBP was degraded by *Bacillus amyloliquefaciens* subsp. strain JR20 within 96 h [56], and strain *Sphingobium yanoikuyae* SHJ degraded 50% of 50 mg/l DBP in 101.4 h [57]. Therefore, *S. acidaminiphila* BDBP 071 can enrich the repertoire of DBP-degrading strains.

Research has shown that co-metabolism with microbial strains provides a faster rate of degradation with stronger effect, making this system more suitable for environmental remediation than single bacterial culture [40, 58]. The coculture of strains CD-9 and BDBP 071 resulted in higher fenvalerate degradation efficiency than either of the single strains (increased by 33.5%), and the level of DBP decreased by 5.21 mg/l in 48 h. The conceivable reason is that strain CD-9 cannot degrade DBP efficiently as DBP restricts the activity of key enzymes in the metabolism of fenvalerate and further inhibits the metabolism process [59]. Strain BDBP 071 possesses efficient DBP degradation ability. After adding it to the degradation system, the accumulated DBP is absorbed by strain BDBP 071 as an energy source, so it reduces the content of DBP and eliminates the inhibitory effect of DBP on the metabolism of fenvalerate, thereby promoting the degradation of fenvalerate. Meanwhile, Tran *et al*. [60] also proved that the cocultivation of microorganisms could effectively degrade organic pollutants and their related metabolites. In addition, Zhao *et al*. [61] reported that *Catellibacterium* sp. CC-5 degraded 83% of fenvalerate within 7 days. In contrast, the cocultivation of strains CD-9 and BDBP 071 degraded 84.37 ± 1.25% of the fenvalerate in 48 h. This indicates that the co-cultivation of strains CD-9 and BDBP 071 can effectively improve the degradation of fenvalerate.

Numerous studies have demonstrated the metabolic mechanisms of single strains. However, there are few reports on the degradation pathways and mechanisms underlying microbial cocultivation. Degradation enzymes play a central role in the bioremediation of pollutants [16]. CD-9 and BDBP 071 coculture strains contain more degradation products than the fenvalerate degradation products generated by strain CD-9 [22] and the DBP degradation products generated by strain BDBP 071. It may be that the cocultivation of the strains produces more enzymes, thereby promoting the degradation of fenvalerate and DBP. Larisa *et al*. [62] found that the coculture of *Citrobacter freundii* S04 and *Sphingobacterium multivorum* W15 significantly increased the enzyme activity. Chen *et al*. [63] showed that compared to monoculture, coculture of *Penicillium citrinum* WXP-2 and *Citrobacter freundii* WXP-9 exhibited the highest microbial activity, which could enhance the degradation of pollutants. However, the mechanism used by coculture strains to promote the degradation efficiency of fenvalerate is not clear and will require additional research on related degrading enzymes. To better understand the mechanism of synergistic strains on the degradation of fenvalerate, ‘omics’ technologies, such as whole genomics, transcriptomics, and metabolomics, will be required in future research.

A DBP-degrading bacterial strain, *Stenotrophomonas* BDBP 071, was isolated and characterized. The optimum conditions for DBP biodegradation were determined to be 30.0 mg/l DBP concentration, initial pH of 7.5, and 37°C culture temperature, which resulted in 88.34 ± 1.44% degradation of DBP within 48 h. Furthermore, cocultivation of strains was used to enhance the degradation of fenvalerate. The concentration of fenvalerate, the inoculation sequence, and the inoculation proportion played important roles in the effective degradation of fenvalerate in coculture of strains CD-9 and BDBP 071. Finally, based on metabolites analysis, a possible biodegradation pathway of fenvalerate was proposed after strain cocultivation. In summary, the study revealed that using cocultures is an efficient approach for removal of PPs and intermediate metabolites from the contaminated environment.

## Supplemental Materials

Supplementary data for this paper are available on-line only at http://jmb.or.kr.

## Figures and Tables

**Table 1:** Analysis of variance (ANOVA) for the fitted quadratic model for DBP biodegradation.

Source	Sum of squares	Degrees of freedom	Mean square	F-Value	*p*-Value
Model	1430.95	9	158.99	48.29	< 0.0001
A-pH	352.58	1	352.58	107.08	< 0.0001
B-Temperature	36.38	1	36.38	11.05	0.0127
C-DBP concentration	39.74	1	39.74	12.07	0.0103
A×B	47.75	1	47.75	14.50	0.0066
A×C	62.17	1	62.17	18.88	0.0034
B×C	127.24	1	127.24	38.64	0.0004
A^2^	0.17	1	0.17	0.053	0.8244
B^2^	648.59	1	648.59	196.98	< 0.0001
C^2^	84.27	1	84.27	25.59	0.0015
Residual	23.05	7	3.29		
Lack of fit	14.20	3	4.73	2.14	0.2382
Pure error	8.85	4	2.21		
Total	1454.00	16			

R^2^ = 0.9841, R^2^_adj_ = 0.9638, *p*-Value < 0.05 indicates that the model terms are significant.

**Table 2:** Identification of intermediate metabolites of DBP using GC-MS.

Serial number	RT(min)	Similarity (%)	Chemical formula	m/z	Name
a	5.180	92	C_4_H_8_O_2_	88	Butyric Acid
b	6.815	65	C_8_H_6_O_2_	134	O-Phthalaldehyde
c	6.820	66	C_7_H_6_O	106	Benzaldehyde
d	9.985	69	C_9_H_8_O_4_	180	Mono-Methyl phthalate
e	15.710	98	C_16_H_22_O_4_	278	Dibutyl phthalate

**Table 3:** Identification of intermediate metabolites of fenvalerate using GC-MS.

Serial number	RT (min)	Similarity (%)	Chemical formula	m/z	Name
A	3.758	97	C_5_H_10_O_2_	102	Isovaleric acid
B	5.075	94	C_7_H_6_O	106	Benzaldehyde
C	5.955	71	C_6_H_6_O	94	Phenol
D	6.815	62	C_8_H_6_O_2_	134	O-Phthalaldehyde
E	6.958	90	C_7_H_14_O_2_	116	4-Methylhexanoic acid
F	11.208	97	C_8_H_8_O_2_	136	Phenylacetic acid
G	13.750	95	C_7_H_7_NO_2_	137	Anthranilic acid
H	16.808	68	C_11_H_13_Cl	212	2-(4-Chlorophenyl)-3-methylbutanoic acid
I	19.775	85	C_13_H_12_O_2_	200	3-Phenoxybenzyl alcohol
J	22.050	98	C_16_H_22_O_4_	278	Dibutyl phthalate
K	33.800	82	C_7_H_6_O_4_	154	Phloroglucinol aldehyde
L	37.883	94	C_25_H_22_ClNO_3_	419	Fenvalerate
