# Supplementary material for: Isolation of Dibutyl Phthalate-Degrading Bacteria and Its Coculture with Citrobacter freundii CD-9 to Degrade Fenvalerate
Source: J Microbiol Biotechnol. 2022 Jan 14;32(2):176–86. doi: 10.4014/jmb.2110.10048 (PMC9628840; doi:10.4014/jmb.2110.10048)
Supplement: Supplementary file 1 [file jmb-32-2-176-supple.pdf]

J. Microbiol. Biotechnol.
https://doi.org/10.4014/jmb.2110.10048

Supplementary Materials

Table S1. Physio-biochemical characteristics of strain BDBP 071.

Characteristic	Result	Characteristic	Result
Gram staining	-	Gelatin liquefaction	-
Catalase	+	M.R test	-
Indole	-	V-P test	+
Sportiness	-	Starch hydrolysis	-
Lysine decarboxylase	-	Ornithine decarboxylase	-
Oxidase	+	Hydrogen sulfide	-

Note: +, tested positive/utilized as substrate; -, tested negative/unutilized as substrate.

Table S2. Optimization of DBP degradation using response surface methodology.

Run	pH	Temperature (°C)	DBP concentration (mg/L)	DBP degradation (%)
1	1	0	-1	76.02 ± 1.78
2	0	1	-1	67.07 ± 2.31
3	0	-1	-1	51.06 ± 1.27
4	0	0	0	78.28 ± 0.95
5	1	-1	0	66.00 ± 1.52
6	-1	-1	0	62.13 ± 1.59
7	1	0	1	87.56 ± 2.26
8	0	0	0	76.22 ± 0.57
9	-1	0	1	63.90 ± 1.78
10	1	1	0	76.71 ± 2.06
11	0	-1	1	67.60 ± 0.86
12	-1	0	-1	68.13 ± 2.72
13	0	0	0	79.15 ± 1.38
14	-1	1	0	59.22 ± 1.64
15	0	0	0	80.21 ± 1.24
16	0	0	0	79.04 ± 0.72
17	0	1	1	61.05 ±1.07

pH: −1(6.5), 0 (7.0), 1 (7.5); temperature (°C): −1 (34), 0(37), 1 (40); DBP concentration (mg/L): −1 (15), 0 (25), 1(35).

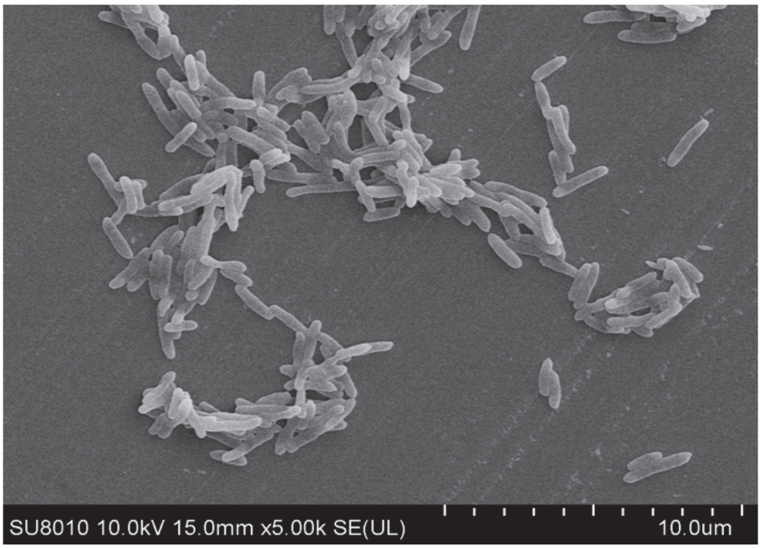

Fig. S1. Morphology of *Stenotrophomonas acidaminiphila* cells under scanning electron microscop

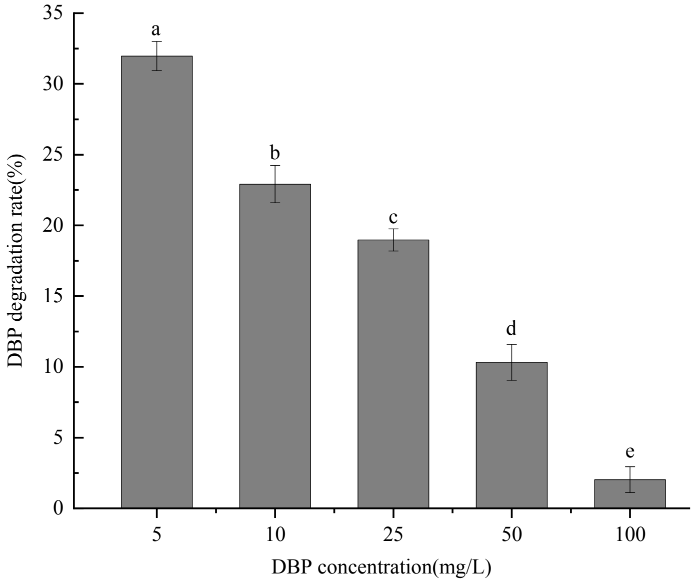

Fig. S2. Degradation of different initial concentrations of DBP by *C. freundi* CD-9. Different letters (a-e) indicate significant differences among treatments ($p < 0.05$).
